# Mucosal administration of α-fodrin inhibits experimental Sjögren's syndrome autoimmunity

**DOI:** 10.1186/ar2403

**Published:** 2008-04-18

**Authors:** Jing He, Jinxia Zhao, Zhanguo Li

**Affiliations:** 1 Department of Rheumatology & Immunology, People's Hospital, Peking University Medical School, 11 Xizhimen South Street, Beijing 100044, China

## Abstract

**Introduction:**

α-Fodrin is an autoantigen in Sjögren's syndrome. We hypothesized that mucosal administration of α-fodrin might prevent the disease.

**Methods:**

Four-week-old NOD mice were immunized (intranasal) with a 1 μg or 10 μg dose of α-fodrin every other day. PBS 10 μl/dose and Glutathione transferase (GST 10 μg/dose (control mice) were intranasally administrated by the same procedure. The salivary flow was maintained in immunized animals. The animals were analyzed for the presence of anti-Sjögren's syndrome A, anti-Sjögren's syndrome B, rheumatoid factor and antinuclear, anti-α-fodrin, and anti-type 3 muscarinic acetylcholine receptor polypeptide (anti-M3RP) by immunofluorescence or ELISA. The cytokines IFNγ and IL-10 were measured by ELISA. Salivary glands were examined by H&E staining and immunohistochemical analysis. The water-volume intake was calculated for each group. The induction of regulatory T cells was assessed by fluorescence-activated cell sorting analysis for the frequency of Foxp3^+ ^cells among peripheral CD4^+^CD25^+ ^T cells.

**Results:**

The appearance of anti-α-fodrin and anti-M3RP antibodies was delayed in mice immunized with α-fodrin. The titers of anti-α-fodrin and anti-M3RP antibodies were lower in immunized mice (*P *< 0.05), but there was no significant difference between the low-dose or high-dose immunization groups. Five out of eight mice in the GST group, five of eight mice in the PBS group, two of eight mice in the α-fodrin 1 μg/dose group, and three out of eight mice in the α-fodrin 10 μg/dose were positive for antinuclear antibodies. The levels of serum IFNγ in mice immunized with 1 μg/dose or 10 μg/dose α-fodrin, with PBS, and with GST were 41.9 ± 16.2 pg/ml, 37.1 ± 15.4 pg/ml, 86.8 ± 17.8 pg/ml and 71.6 ± 11.1 pg/ml, respectively, while we found no difference in the levels of serum IL-10 among the groups. The number of Foxp3^+ ^CD4^+^CD25^+ ^regulatory T cells was higher in the α-fodrin groups compared with the PBS and GST control groups (*P *< 0.05). Lymphocytic infiltration and expression of α-fodrin in the salivary glands was decreased in α-fodrin-treated groups. The fluid intake of mice in the 1 μg/dose α-fodrin, 10 μg/dose α-fodrin, PBS, and GST groups was 39.2 ± 2.1 ml, 40.4 ± 2.5 ml, 49.3 ± 3.1 ml and 51.6 ± 2.8 ml, respectively.

**Conclusion:**

Mucosal administration of α-fodrin effectively inhibited the progression of experimental Sjögren's syndrome autoimmunity.

## Introduction

Primary Sjögren's syndrome (SS) is a chronic autoimmune disorder of unknown etiology. Lymphocytic infiltration of the lachrymal and salivary glands leads to dry mouth (xerostomia) and dry eyes (xerophthalmia). It has been assumed that a combination of immunologic, genetic, and environmental factors plays an important role in the development of autoimmune abnormalities in primary SS.

In 1997 Haneji and colleagues identified a 120 kDa fragment of the ubiquitous cytoskeletal protein α-fodrin as an autoantigen in the NFS/sld mouse model of human SS [1]. It has been shown by immunoblotting that anti-α-fodrin antibodies are present in sera from 93% of primary SS patients and from 63% of secondary SS patients, but not in sera from systemic lupus erythematosus (SLE) patients or rheumatoid arthritis patients and normal control individuals [1]. In a previously published study we reported similar results, finding that these antibodies are positively correlated with the systemic manifestation of SS [2,3]. *In vivo *roles of α-fodrin N-terminal portion peptides were investigated using peripheral blood mononuclear cells from patients with SS, from patients with SLE, and from patients with rheumatoid arthritis. Significant proliferative T-cell responses of peripheral blood mononuclear cells o α-fodrin peptide were detected in SS but not in SLE or rheumatoid arthritis [4]. We previously identified *in vivo *immunoregulation of α-fodrin using the same method. Kurien and colleagues had induced oral tolerance in experimental SS animal successfully [5]. There have been other reports of nasal and oral tolerance in SS [6,7], a phenomenon that brings about systemic immune hyporesponsiveness by the exogenous administration of antigen to the peripheral immune system through the mucosal route.

We hypothesized that nasal tolerance could be induced in an experimental animal model of SS by nasal administration of α-fodrin, potentially preventing and inhibiting the development of SS. The present study was undertaken in order to test this hypothesis.

## Materials and methods

### Materials

The plasmid (pGEX-4T-2–α-fodrin) was a generous gift from Professor Hayashi of the Tokushima University School of Dentistry in Japan.

### Large-scale bacterial expression and fusion protein purification

A 5 ml saturated culture of pGEX-4T-2–α-fodrin-transformed bacteria grown in Luria-Bertani(LB) medium supplemented with ampicillin (100 μg/ml) was used to inoculate 200 ml LB medium. This culture was allowed to reach the log phase (*A*_600 _= 0.6) before induction with Isopropyl β-D-1-Thiogalactopyranoside (IPTG) (1 mM final). The induction was carried out for 4 hours at 37°C (300 rpm), and then the bacteria were harvested by centrifugation (1,200 × *g *for 10 min, 4°C). The resulting pellets were resuspended in 24 ml ice-cold 10 mM Tris (pH 7.5), 10% glycerol, 10 mM dithiothreitol. Bacteria were lysed by sonication, and bacterial inclusion bodies were collected by centrifugation (40,000 × *g *for 10 min, 4°C). The sample was passed over a 5 ml glutathione-Sepharose affinity column equilibrated with PBS, and the fusion protein was eluted with 10 ml elution buffer (5 mM glutathione, 50 mM Tris, pH 8.0) collected in 2 ml fractions. The final yield of purified α-fodrin was 2 mg.

### Mice

Female NOD mice (4 weeks old) were purchased from the Laboratory Animal Resource Center of the Peking University Medical Science Center and were acclimatized to the center for 1 week before starting the experimental protocol. Animals were maintained in a specific pathogen free environment, and were fed standard rodent chow and water *ad libitum*. The study was approved by the Animal Care and Use Committee of the Peking University People's Hospital.

### Treatment protocol

Four groups, each consisting of eight NOD mice, were used. Groups I and II were immunized every other day with 1 μg and 10 μg.

Glutathione transferase–α-fodrin fusion protein, respectively. Group III was immunized with 10 μg Glutathione transferase every other day, and Group IV was immunized with 10 μl PBS every other day. Immunization was given by nasal administration. Preimmune bleeds were collected from all mice. Postimmune bleeds were collected every 2 weeks. Mice were killed at 17 weeks of age by cervical dislocation.

### Peptides

The sequence of the type 3 muscarinic acetylcholine receptor second loop polypeptide (KRTVPPGECFIQFLSE) is a known M3R epitope based on previous studies [5]. This linear peptide was synthesized using solid-phase techniques on an Applied Biosystems Peptide Synthesizer (APEX396, AAPPPEC, Kentucky, USA) at SBS Gene Technology Company (Shanghai, China). The peptide was purified by reversed-phase high-performance liquid chromatography to a purity >90%.

### Quantitation of anti-type 3 muscarinic acetylcholine receptor polypeptide antibody by ELISA

A solid-phase immunoassay for the type 3 muscarinic acetylcholine receptor polypeptide (M3RP) was performed as previously described. Briefly, 96-well ELISA plates (Costarvinyl, Cambridge, MA, USA) were coated with 100 μl of 10 μg/ml M3RP in 0.5 M carbonate buffer, pH 9.6. Coating was performed at 4°C overnight, followed by blocking with 100 μl of 5% milk in PBS (pH 7.4) containing 0.1% BSA. After washing with PBS–Tween 20, the wells were then loaded with 100 μl mouse serum (1:100 dilution) and were incubated at room temperature for 2 hours, at which point the wells were washed five times with PBS–Tween 20. Then 100 μl goat anti-mouse IgG conjugated to peroxidase (1:4,000; Zhongshan Technology Company, Beijing, China) was added to each well and incubated for 1 hour at room temperature. After washing five times with PBS–Tween 20, the bound antibodies were detected with the substrate, *O*-phenylenediamine. The reaction was stopped by addition of 100 μl of 2.5 M sulfuric acid to each well. Plates were read at a wavelength 492 nm (absorbance, OD_492 nm_) with an ELISA plate reader (BIO-RAD Model 550; MICROPLATE READER, Hercules California, USA). Each serum sample was assayed in duplicate. The results were expressed as optical density units ± standard deviation. An optical density value greater than two standard deviations from the mean optical density value of the control sera was considered positive.

### Autoantibody quantitation

The IgG class autoantibodies against SSA, SSB, rheumatoid factor (Euroimmun, Lübeck, Germany) and anti-α-fodrin antibody (AESKI, Wendelsheim, Germany) were measured by ELISA. Antinuclear antibodies were measured by immunofluorescence according to the manufacturer's instructions (Euroimmun).

### Preparation of lymphocytes and flow cytometry

Lymphocyte suspensions were prepared from spleens and lymph nodes. For flow cytometric analysis, 1 × 10^6 ^cells were incubated with fluorescein isothiocyanate-labeled anti-CD4 and activated protein C-labeled anti-CD25 (BD Biosciences, Franklin Lakes, NJ USA). For intracellular staining of Foxp3, lymphocytes were first surface-labeled (CD4 and CD25), were then fixed and permeabilized using the Cytofix/Cytoperm kit (BD Biosciences), and finally were stained with anti-Foxp3-PE (BD Biosciences). Cells were analyzed by flow cytometry (BECTONDICKINSON, FACSort, Franklin Lakes, NJ USA).

### Analysis of cytokine production

Samples of blood were collected at the end of the experiment. Serum levels of IFNγ and IL-10 were measured with a commercial ELISA kit (R&D, Minneapolis, Mn. USA) according to the recommendations of the manufacturer.

### Histological and immunohistochemical analysis

After the mice were killed, the submandibular and sublingual salivary glands were surgically removed, fixed in 10% buffered formalin, and embedded in paraffin. Four-micrometer sections were prepared. H&E staining was performed to determine the degree of inflammation. Infiltrates appear as periductal and perivascular foci within the glandular architecture of the salivary glands. Focal scores classified as 1 or above, which consist of clusters of >50 lymphocytes in a 4 mm^2 ^area, are considered abnormal. Immunostaining was performed by the avidin–biotin complex method, using anti-α-fodrin polyclonal antibody (Cell Signaling, Danvers, Boston, USA).

### Measurement of the water volume intake of mice

We fed and watered the mice carefully. Each bottle of water was measured to 200 ml on the first weekday, and the remaining water volume at the end of the week was measured to calculate the volume of water drunk by the mice. Food was given to the mice separate from the water. Mice were separated and the volume of water was measured every week. The volumes of the groups were then compared.

### Statistical analysis

Experimental findings were presented using the Mann–Whitney U test (two-tailed, independent). Analyses were carried out using SPSS 13.0 software (SPSS^® ^Base 13.0, Wacker Drive, Chicago, USA). The statistical significance level was set at *P *= 0.05.

## Results

### Effect of α-fodrin immunization on autoantibody production

**Figure 1:**
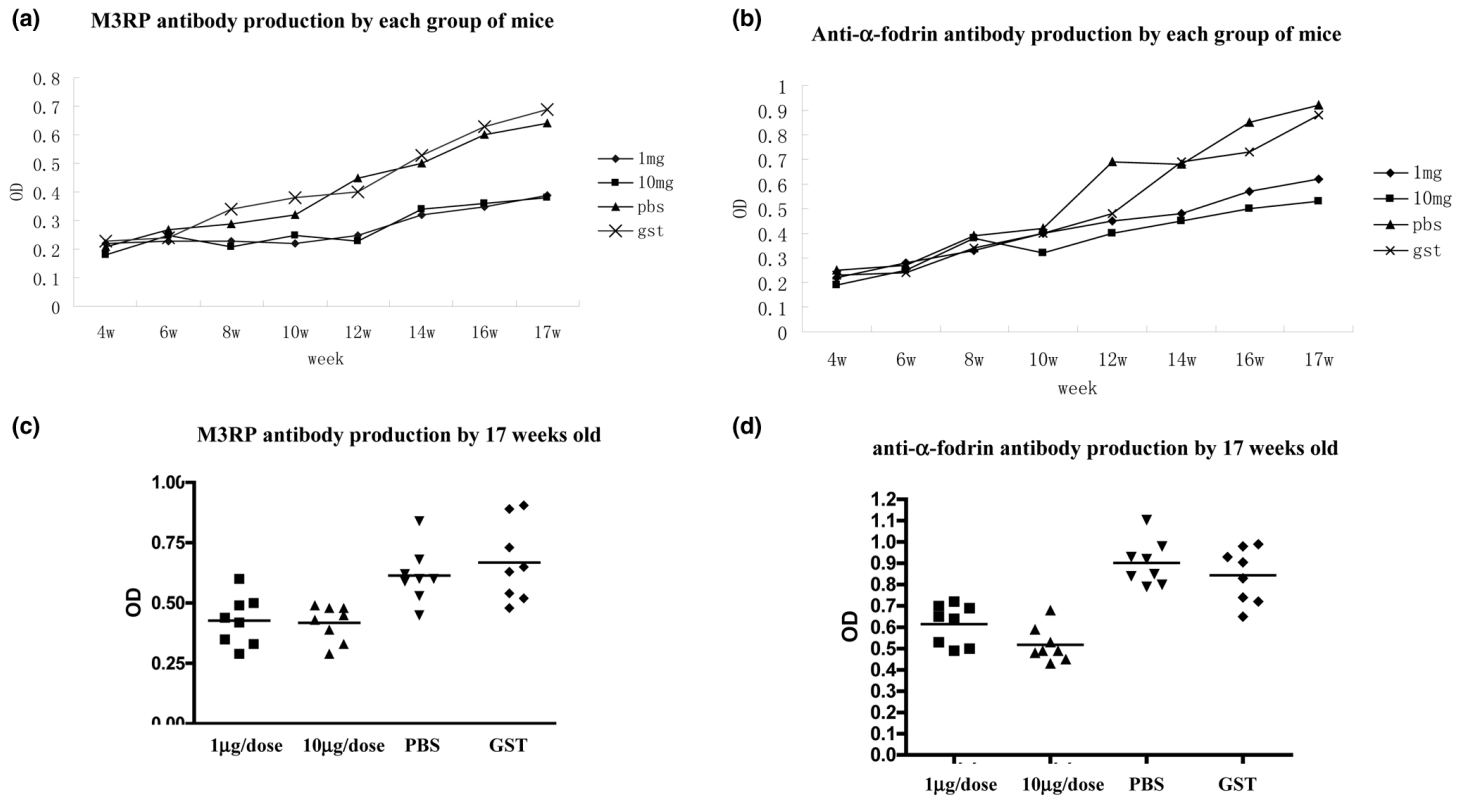
**α-Fodrin immunization effect on type 3 muscarinic acetylcholine receptor polypeptide antibody and anti-α-fodrin antibody production**. Titer of **(a) **anti-type 3 muscarinic acetylcholine receptor polypeptide (anti-M3RP) antibodies and **(b) **anti-α-fodrin antibodies increased in each group of mice with time. By 17 weeks of age, the titer of **(c) **anti-M3RP antibodies and **(d) **anti-α-fodrin antibodies was lower in mice immunized with α-fodrin (*P *< 0.05), but there was no significant difference between the two α-fodrin-immunized groups. Glutathione transferase; OD, optical density.

To detect any effect of α-fodrin immunization of NOD mice, we analyzed the appearance of anti-M3RP, anti-α-fodrin, anti-SSA and anti-SSB antibodies, as well as rheumatoid factor and antinuclear antibodies. As shown in Figure 1, the titer of anti-α-fodrin and anti-M3RP antibodies was lower in mice immunized with α-fodrin (*P *< 0.05), but there was no significant difference between the two α-fodrin dosage groups. Five out of eight mice in the GST control group, five of eight mice in the PBS group, two of eight mice in the 1 μg/dose α-fodrin group, and three out of eight mice in the 10 μg/dose α-fodrin group were positive for antinuclear antibodies (all with speckled pattern). Antibodies against SSA and SSB, and against rheumatoid factor, however, were not detected in any of the mice.

These results demonstrate that intranasal administration of α-fodrin can suppress the production of SS-related antibodies, while PBS or GST treatment had no dramatic effect on autoantibody production.

### Effect of α-fodrin immunization on cytokine production

**Figure 2:**
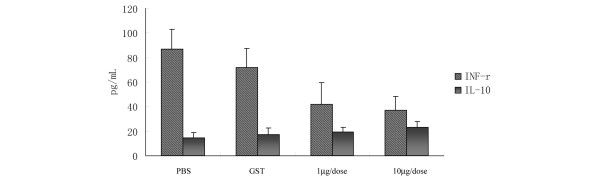
**Effect of α-fodrin immunization on cytokine production**. Serum levels of IFNγ in mice in the 1 μg/dose α-fodrin, 10 μg/dose α-fodrin, PBS, and Glutathione transferase groups were 41.9 ± 16.2 pg/ml, 37.1 ± 15.4 pg/ml, 86.8 ± 17.8 pg/ml, and 71.6 ± 11.1 pg/ml, respectively. IFNγ levels of the α-fodrin groups were much lower than those of the control groups (*P *< 0.05), but no difference was found of the levels of serum IL-10 among the four groups.

The serum levels of IFNγ and IL-10 were detected by ELISA, as described in a this study. The serum levels of IFNγ in mice immunized with the 1 μg/dose or 10 μg/dose α-fodrin, with PBS, and with GST were 41.9 ± 16.2 pg/ml, 37.1 ± 15.4 pg/ml, 86.8 ± 17.8 pg/ml, and 71.6 ± 11.1 pg/ml, respectively (Figure 2). The levels of IFNγ in the α-fodrin groups were much lower than those in control mice (*P *< 0.05), but there was no significant difference in the levels of serum IL-10 among the four groups. This result suggested that nasal administration of α-fodrin was able to prevent the *in vivo *production of the inflammatory cytokine IFNγ, but had little influence on the production of the anti-inflammatory cytokine IL-10.

### Effects of α-fodrin immunization on Foxp3^+ ^CD4^+^CD25^+ ^regulatory T cells

**Figure 3:**
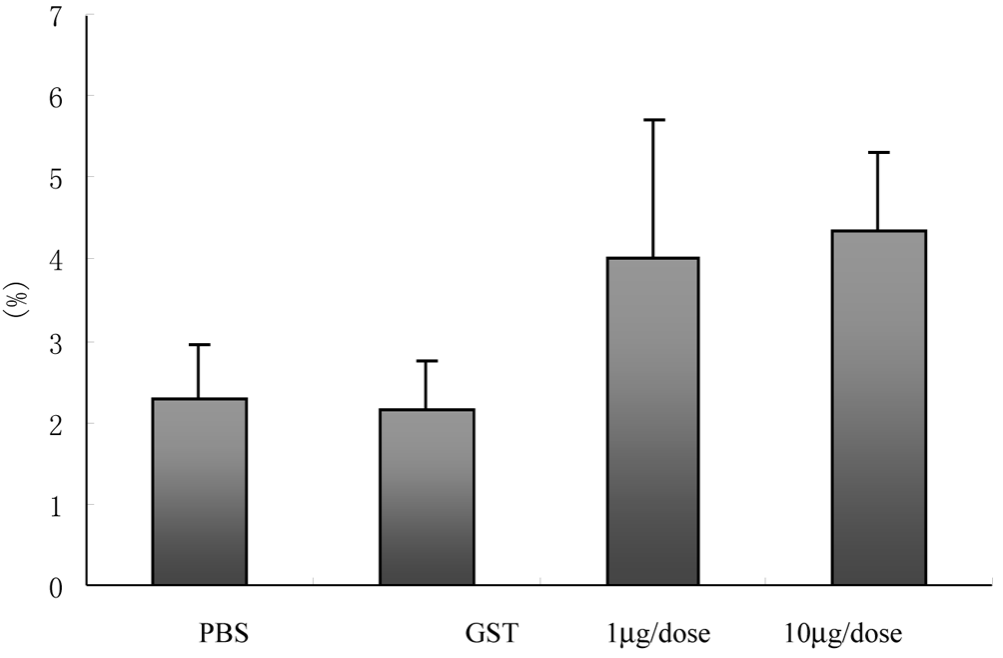
**Effect of α-fodrin immunization on Foxp3^+ ^CD4^+^CD25^+ ^regulatory T cells**. Frequency of Foxp3^+ ^CD4^+^CD25^+ ^regulatory T cells was higher in the α-fodrin groups than in the PBS and Glutathione transferase control groups (*P *< 0.05), but there was no difference between the two fodrin groups.

As shown in Figure 3, the frequency of Foxp3^+ ^regulatory T cells among the CD4^+^CD25^+ ^T-cell subset in the mice treated with 1 μg/dose α-fodrin, 10 μg/dose α-fodrin, PBS, and GST were 4.01 ± 1.70%, 4.34 ± 0.98%, 2.17 ± 0.60%, and 2.29 ± 0.66%, respectively. The number of Foxp3^+ ^CD4^+^CD25^+^regulatory T cells was higher in the fodrin groups compared with the PBS and GST controls (*P *< 0.05), but there was no difference between the 1 μg/dose and 10 μg/dose α-fodrin groups.

### Effects of α-fodrin immunization on the histopathologic analysis of NOD mice

**Figure 4:**
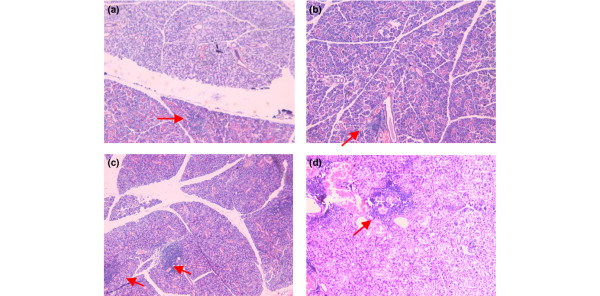
**Effect of α-fodrin immunization on the histopathologic analysis of NOD mice**. Effect of α-fodrin immunization on the histopathologic analysis in the **(a) **1 μg/dose α-fodrin group, **(b) **10 μg/dose α-fodrin group, **(c) **PBS group, and **(d) **Glutathione transferase group. Tissues were stained with H&E. Lymphocytic infiltration was decreased in the glands of α-fodrin-administrated groups ((a) and (b) Each red arrow represent the focus of the lyphocytic infiltration.).

**Figure 5:**
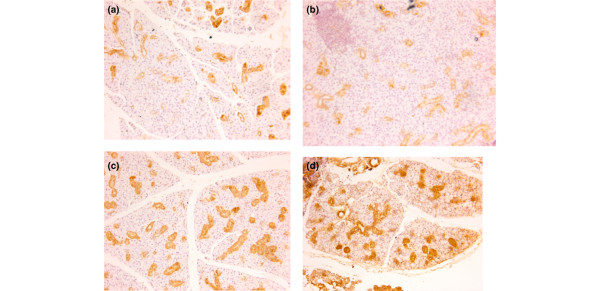
**Effect of α-fodrin expression on histopathologic analysis of NOD mice**. Effect of α-fodrin expression on histopathologic analysis in the **(a) **1 μg/dose α-fodrin group, **(b) **10 μg/dose α-fodrin group, **(c) **PBS group, and **(d) **Glutathione transferase group. Immunohistochemical analysis of salivary gland sections from NOD mice using polyclonal antibodies against α-fodrin. Epithelial duct cells were intensely stained, especially within the inflammatory lesions. Immunoreactivity was decreased in the salivary glands of α-fodrin-administrated groups ((a) and (b)).

As shown in Figure 4, the sublingual salivary glands of each mouse were infiltrated by lymphocytes, and seem to be abnormal. As shown in Figures 4 and 5, however, lymphocytic infiltration and expression of α-fodrin was decreased in the salivary glands of α-fodrin-immunized groups. These results show that nasal administration of α-fodrin can suppress destruction of the salivary gland, while the GST and PBS control treatments failed to interfere with an ongoing SS process.

### Effects of α-fodrin immunization on water volume intake of NOD mice

**Figure 6:**
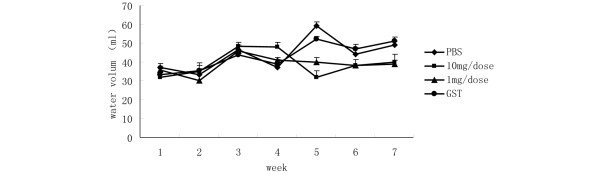
**Effect of α-fodrin immunization on water volume intake of NOD mice**. Mice were monitored for water consumption until 17 weeks old. Mice from the 1 μg/dose α-fodrin, 10 μg/dose α-fodrin, PBS, and Glutathione transferase groups showed water consumption of 39.2 ± 2.1 ml, 40.4 ± 2.5 ml, 49.3 ± 3.1 ml, and 51.6 ± 2.8 ml, respectively. The volume of fluid intake by α-fodrin-immunized groups was therefore lower than in the control groups (*P *< 0.05).

As shown in Figure 6, at the end of the experiment, when the mice were 17 weeks old, the volumes of water consumed per mouse in the 1 μg/dose α-fodrin, 10 μg/dose α-fodrin, PBS, and GST groups were 39.2 ± 2.1 ml, 40.4 ± 2.5 ml, 49.3 ± 3.1 ml, and 51.6 ± 2.8 ml, respectively. The volume for α-fodrin groups was significantly lower than that for the control groups (*P *< 0.05). These results show that nasal administration of α-fodrin can maintain function of the salivary gland.

## Discussion

There has been much investigation into the different aspects of the pathogenesis behind human autoimmune diseases, including SS. The hypofunction of the salivary and lachrymal glands characteristic of SS is due to lymphocytic infiltration. It is generally assumed that autoreactive T cells recognize an unknown self-antigen and play a central role in the pathogenesis of this disease.

Various autoantibodies reactive with epitopes expressed in target organs have been detected in the sera from patients with autoimmune disease [1,8-11]. These antibodies – directed against nuclear, cytoplasmic, and cell-surface proteins – have been used as diagnostic tools for disease onset and progression [1,12,13]. One of the first-described antibodies associated with autoimmune diseases was antinuclear antibodies, and then SSA and SSB antibodies were found, especially in SS. Other sets of autoantigens appear to be generated during the process of apoptosis through the exposure of potential cryptic antigens following cleavage by activated caspases. For example, the proteolytic product of autoantibodies in SS is of potential diagnostic value [1], although the role in disease initiation or progression has yet to be defined.

Fodrin is an actin-binding protein in the cortical cytoskeleton of most eukaryotic cells that is associated with autoimmune lesions in thymectomized mice [1,14]. Our previous studies have shown that α-fodrin antibodies can be detected in the sera of most primary SS patients and are positively correlated with the extrasecretory manifestations [2,3,15]. A study by Haneji and colleagues suggested that serum from an NFS/sld mouse model of human SS reacted with α-fodrin, specifically expressed in the lesional salivary glands [1]. Using peripheral blood mononuclear cells from patients with SS, from patients with SLE, and from patients with rheumatoid arthritis, Miyazaki and colleagues reported that significant *in vitro *proliferation of T cells in response to α-fodrin was detected in SS, but not in SLE and rheumatoid arthritis [4]. All these studies showed the role of autoantigen in the development of primary SS.

Studies on the NOD mouse have begun to provide us with invaluable insights into the pathogenic mechanisms underlying the development of SS. The NOD mouse revealed the existence of two phases in the pathogenesis of SS. First, there is the disruption of homeostasis in the target tissue, resulting in increased apoptosis as well as cysteine proteinase activity, aberrant protein expression, and loss of amylase activity. Second, the involvement of immune components leading to activation of T cells and B cells and the generation of autoantibodies provide the hallmark clinical symptoms of dry eyes and dry mouth. Studies have, however, shown that only 10% of NOD mice can be detected as anti-SSA-positive [16,17]. There had been over 60 mice used in our previous study and the present study, but no NOD mouse studied presented anti-SSA antibody in serum – which may related to the mice we bought and also to the methods used to detect the antibodies. As we know, NOD mice can also be used as a diabetic model. In our study, the blood sugar of two mice in each group elevated by the end of 16 weeks, so all mice were sacrificed soon after to avoid the influence of possible diabetic disorder.

Generally, immune tolerance is acquired by various mechanisms during maturation of the immune system. Low doses of administered antigen favor active suppression [18]. Nasal administration of autoantigens has been used to treat autoimmune diseases in animals and in humans [19,20]. In the present study, we tried to identify whether nasal tolerance could be induced in an experimental animal model of SS by mucosal administration of α-fodrin, thus preventing disease development. Mucosal tolerance prevents the body from eliciting productive immune responses against harmless antigens that enter the body via the mucosal route, and is mediated by the induction of regulatory T cells that differentiate in the mucosa-draining lymph nodes under defined conditions of antigen presentation [6,7].

In the present study, we noticed that the number of Foxp3^+ ^CD4^+^CD25^+ ^regulatory T cells was higher in the α-fodrin groups than in the PBS and GST control groups, the amount of water consumed by mice of the α-fodrin groups was lower, and the titer of SS-related antibodies was lower. These events may initiate local IgG in the mucosa-draining lymph nodes, downregulating dendritic cell activation induced by nasally applied antigen – resulting in those defined conditions of antigen presentation that lead to regulatory T-cell induction and tolerance; however, this pathway still needs to be further identified.

Previous reports show that these mice could be measured for salivary flow directly, but we are less skilled in this area and cannot measure the salivary flow precisely. So water consumption was calculated to measured salivary flow.

We show here that SS-related antibodies in the serum and the amount of water consumed can be decreased by mucosal administration of low doses of α-fodrin. In addition, this protein can prevent or limit lymphocyte infiltration, as well as the expression of α-fodrin antigen in the salivary glands, which may play a role in the salivary flow decrement. Although we have not seen a clear relevance of salivary flow with the amount of water consumed and with the antibodies apparent in the serum, it is possible that antibodies such as anti-M3RP and anti-α-fodrin antibodies may have a role in the salivary flow decrement.

The mechanism by which this treatment protects against impaired gland activity has not yet been elucidated. We propose that multiple low doses of autoantigen may result in the generation of antigen-specific regulatory cells, which in turn secrete immunosuppressive cytokines such as transforming growth factor beta upon restimulation with the antigen. Our results indicated that the number of Foxp3^+ ^CD4^+^CD25^+^regulatory T cells was higher in the α-fodrin groups than in the PBS and GST control groups, which supports this idea. In addition, the Th1/Th2 balance may also contribute to the processes of disease development or tolerance. As shown above, the Th1 cytokine IFNγ was increased in the α-fodrin immunized group, while IL-10, a Th2 cytokine, was not. Even though we failed to detect a significant increase of IL-10, the role of Th2 cytokines cannot be entirely ruled out, particularly for cytokines such as IL-10 and transforming growth factor beta, which may play a role.

Following their generation, antigen-specific regulatory cells then migrate to the lymphoid organs where they can suppress immune responses by inhibiting the generation of effecter cells. They may also migrate to the target organ and suppress disease by releasing cytokines. We superficially investigated the regulatory mechanisms controlling SS in this model, but further studies will be required.

In conclusion, we were able to demonstrate that nasally administrated α-fodrin can induce tolerance against this antigen, blocking the lymphocytic infiltration of the salivary gland, and influencing salivary dysfunction in this animal model of SS.

## Conclusion

Mucosal administration of α-fodrin effectively suppresses the production of SS-related antibodies, prevents the *in vivo *production of inflammatory cytokines, such as IFNγ, and increases the number of Foxp3^+ ^CD4^+^CD25^+ ^regulatory T cells. The present study raised the hypothesis that mucosal administration of α-fodrin possibly inhibits the progression of experimental SS autoimmunity.

## Abbreviations

BSA = bovine serum albumin; ELISA = enzyme-linked immunosorbent assay; GST = Glutathione transferase; H&E = hematoxylin and eosin; IFN = interferon; IL = interleukin; M3RP = type 3 muscarinic acetylcholine receptor polypeptide; PBS = phosphate-buffered saline; SLE = systemic lupus erythematosus; SS = Sjögren's syndrome; Th = T helper.

## Competing interests

The authors declare that they have no competing interests.

## Authors' contributions

Jing He performed most of the experiments. Jingxia Zhao performed experiments in animal test. Zhanguo Li conceived the study and participated in the design, in the interpretation of results. Jing He participated in drafting the manuscript. All authors read and approved the final manuscript.
